# Impact of cessation of caffeine citrate therapy on intermittent hypoxemia patterns among preterm infants born before 34 weeks

**DOI:** 10.3389/fped.2025.1463484

**Published:** 2025-06-23

**Authors:** Peter Kipkurui Mashep, Roseline Ochieng, Jesse Coleman, Mary Waiyego, Amy Sarah Ginsburg, Dorothy Chomba, Morris Ogero Ondieki, Grace Irimu, William M. Macharia, J. Mark Ansermino

**Affiliations:** ^1^ Department of Paediatrics, Aga Khan University, Nairobi, Kenya; ^2^ Department of Medicine, University of British Columbia, Vancouver, BC, Canada; ^3^ Department of Paediatrics, Kenyatta National Hospital, Nairobi, Kenya; ^4^ Clinical Trials Center, University of Washington, Seattle, WA, United States; ^5^ Department of Infectious Disease Epidemiology and International Health, London School of Hygiene and Tropical Medicine (LSHTM), London, United Kingdom; ^6^ Department of Paediatrics and Child Health, University of Nairobi, Nairobi, Kenya; ^7^ Department of Anaesthesia, Pharmacology and Therapeutics, University of British Columbia, Vancouver, BC, Canada

**Keywords:** intermittent hypoxemia, caffeine citrate, interrupted time series, preterm infants, LMIC (low and middle income countries)

## Abstract

**Introduction:**

Intermittent hypoxemia (IH) is defined as oxygen saturation (SpO_2_) drop ≥5% from the baseline (set at 90 s preceding the event) to a level less than 90% lasting for ≥5 s. Caffeine citrate, the standard of care for apnea of prematurity, reduces IH events. IH contributes to both short and long-term adverse neurologic outcomes. Standard patient monitors cannot detect IH events due to long averaging times.

**Objective:**

Describe change in patterns of IH events in preterms <34 weeks before and after cessation of caffeine citrate and factors associated.

**Methods:**

Interrupted time series study design. Data was collected from 1 December 2022 to 30 June 2023. MASIMO RAD-G oximeter was used, and analysis done using Trace software V3028 output was desaturation frequency, duration, and time. Data exported, stored, and analyzed using Excel 2016. Change in slope compared visually in two time periods interrupted at 34 weeks and objective statistical analysis done using the Student-*T*-test, CI 95% with *p*-value <0.05 considered significant.

**Results:**

49 patients medical records available for secondary analysis. Frequency of IH events increased from 7.94 to 40.94 events/hour (5-fold). IH events of durations lasting between 0 and 10 s, and >20 s decreased by 12.3% and 6.8%, respectively, while those lasting 10–20 s increased by 17%. The mildly severe IH events decreased by nearly half, 46.9% (78.4% to 31.5%), while both the moderately severe and severe IH events increased by 17.4% (18.5%–35.9%), and 26% (6.7% to 32.7%) respectively. The time spent in hypoxemia increased by 2.3 h/week/patient, while the cumulative time in hypoxemia increased by 1.6 h/patient. Preterms exposure to ACS (antenatal corticosteroids) was associated with decrease in IH events.

**Conclusion:**

Caffeine citrate cessation leads to worsening of IH events with increased frequency, duration, severity and cumulative time spent in hypoxemia. Exposure to ACS was associated with decrease in IH events.

**Recommendation:**

Caffeine citrate therapy use beyond 34 weeks is likely to be beneficial especially in the context of LMIC where antenatal steroid is not always administered, and monitoring of preterm babies is suboptimal. Safe cessation of caffeine therapy requires monitoring to detect IH events.

## Introduction

Intermittent hypoxemia (IH) events are brief repetitive cycles of desaturation (SpO_2_ <90%) followed by a return to normoxemia (SpO_2_ ≥90%) (1). Apnea of prematurity (AoP) and immature control of breathing are the postulated underlying physiological mechanisms in preterm infants (1, 2). Accurate assessment of IH requires continuous pulse oximetry monitoring equipment with short averaging times that display trends (2, 3). Conventional critical care charts do not include IH events which may occur in regular patterns or irregular clusters with variable nadirs (4–6).

Infants with high-risk patterns of IH are predisposed to greater mortality and morbidity, including neurodevelopmental impairment with decreased cognitive development and language delay, retinopathy of prematurity, poor weight gain, and BPD (7–14). Early onset IH is associated with an increased risk of asthma at 2 years of age, while a higher frequency of IH events after 3–5 weeks of age is associated with retinopathy of prematurity (10–13, 15). An increase in IH events can provide early warning to adverse events, such as neonatal sepsis, BPD and prolonged newborn intensive care unit stay (7, 14, 16–18).

The incidence of IH increases with severity of prematurity and among extremely preterm infants born less than 28 weeks gestation, it is lowest immediately after birth steadily increasing over the first 3 days, and peaks between 4 and 6 weeks postnatal age (6, 9, 15). Interventions that reduce the incidence of IH events include caffeine citrate, oxygen supplementation, optimization of respiratory support, and correction of anemia. Caffeine reduces the number of IH events per hour by 52% and results in a reduction in time spent by infants with a SpO_2_ <90% by 47% (seconds spent in hypoxemia per hour monitored) (6, 15, 19–21). While guidelines recommend cessation of caffeine therapy at 34 weeks based on the reduced risk of AoP after this age, there is some evidence that IH events may increase after cessation (19, 21–24).

Challenges facing preterm infants care in low- and middle-income countries include inadequate administration of antenatal corticosteroids (ACS), inconsistent availability and high cost of caffeine citrate, higher prevalence of neonatal co-morbidities (25). In addition, there is insufficient respiratory support and monitoring equipment and inadequately staffed units (25–27). Adequate provision and implementation of antenatal care for anticipated preterm deliveries and caffeine citrate for infants born less than 34 weeks gestation become the cornerstone for improving neonatal outcomes in such settings (21, 26, 27).

## Objectives

The primary objective was to study the impact of cessation of caffeine citrate therapy on the trend of IH events in preterm infants. Secondary objectives were to describe the change in frequency, duration, and severity of IH events, and the time spent in hypoxemia and comparison of IH events in babies exposed and those not exposed to antenatal steroids.

## Methods

### Study site

Nested within a quality improvement study evaluating the feasibility of management of AoP with caffeine citate which took place in the newborn unit (NBU) of a tertiary care teaching hospital in Kenya. Infants were recruited from May 2022 to June 2023, and the IH data collected during phase 2 of the study between December 2022 and June 2023 (25). The study had two phases and provided caffeine with continuous monitoring as the intervention. The first phase consisted of formative research to develop a context-appropriate prototype clinical care bundle for management of AoP that included caffeine citrate and an implementation strategy using currently available evidence, key stakeholders’ consensus opinion, and high-quality context-appropriate clinical data. The second phase was a quality improvement study to pilot implementation and optimization of an AoP clinical care bundle that incorporates caffeine citrate using a plan-do-study-act framework. The quality improvement strategy focused on adoptability, feasibility, usability, acceptability, adherence, and accessibility by target users, including healthcare administrators, healthcare providers, and caregivers of neonates.

### Study population

The eligibility criteria for recruitment to the primary study included all newborn infants admitted to the tertiary NBU during the study period who were less than 34 weeks gestation by dates or had a birth weight of less than 1,500 g and estimated gestational age by the New Ballard score of less than 34 weeks. Eligible neonates whose caregiver provided written informed consent were enrolled and caffeine citrate therapy was based on existing hospital guidelines.

### Study definitions

IH event - a drop in SpO_2_ of at least 5% from baseline with a nadir <90% lasting for at least 5 s (1). Time window for baseline was set at 90 s preceding the event. Desaturation index (DSI) - mean number of IH events per hour recorded (1). Duration of IH events - mean duration in seconds and divided into three categories: 0–10 s; 10–20 s; and >20 s (1).

### Study design

The study design consisted of an interrupted time series (ITS) without a control arm to compare the trend of IH events during and after cessation of caffeine treatment. The intervention was caffeine citrate cessation, and the interruption point was at 34 weeks gestational age. ITS modeling algorithms were used to model the forecast trend in the absence of the intervention (20).

### Study procedures

After obtaining written informed consent, eligible infants were enrolled and started on caffeine citrate based on existing hospital protocols. Treatment decisions for the infants were made by the attending hospital physicians and the dose of caffeine citrate was a standard low-dose regimen with a loading dose of 20 mg/kg administered enterally or parentally at birth followed by 5–10 mg/kg/day maintenance dose as recommended till 34 weeks gestational age. Treatment was stopped once infants ceased having clinically detectable apnea. Occurrence of apnea was monitored clinically by observation for cessation of breathing for more than 20 s, desaturation to less than 90%, and bradycardia of less than 100 beats per minute using the standard patient monitors which were used for clinical decisions. MASIMO Rad G pulse oximetry monitoring devices were applied to eligible infants for durations exclusively determined by the NBU attending physician and were set to provide readings at an averaging time of 2 s intervals. These recordings were stored in the device memory and transferred to the study computer every 24 h. Therefore, not all infants had pulse oximetry recordings from the study devices. Respiratory support options in the unit included blended and unblended humidified oxygen by nasal prongs, continuous positive airway pressure (CPAP), and conventional mechanical ventilation.

Enrolled neonates were followed up from initiation of caffeine treatment up to 7 days after the discontinuation of caffeine as determined by the NBU attending physician or to 38 weeks gestational age. Data collection continued for neonates who underwent escalation of care to CPAP or mechanical ventilation.

### Data management

#### Data collection and storage

Clinical data were collected using REDCap electronic data collection forms and stored in the REDCap software server account. Pulse oximetry recordings were downloaded weekly and stored in the MASIMO Trace V3025, Build Number 028 software database installed in a secure dedicated study laptop.

#### Data analysis

The baseline data, socio-demographic, clinical, morbidity, and treatment characteristics were presented in a summary table. Pulse oximetry data were downloaded from the MASIMO RAD G devices onto the study laptop directly and processed using the MASIMO Trace V3025, Build Number 028 software database tools. Pre-processing was done to crop out blank and invalid sections of records and then valid segments were analyzed in segments of 24-h periods. The Trace software report-generating module produced summaries which were exported as PDF files.

The raw daily data were read and tabulated in a master Microsoft Excel (2016) spreadsheet for each patient and by postmenstrual age. Time series trends were then plotted from the daily data using the Excel chart features for the actual data. The interrupted time series (ITS) analysis was done using autoregressive integrated moving averages (ARIMA) and the Seasonal ARIMA (SARIMA) algorithms where applicable. Between 3 and 5 models were generated using different parameters and the optimal model selected was one with the least Akaike's Information Criterion (AIC) and Bayesian Information Criterion (BIC) (28). The Real Statistics Resource Pack software (Release 8.9.1), Copyright (2013–2023) Charles Zaiontz. https://www.real-statistics.com was utilized.

Aggregate weekly data for actual and forecast model data was done. Interrupted time series charts for each of the study endpoints were then plotted using the aggregated actual and forecast model data to produce the actual and counterfactual trends respectively and the post-interruption slope difference between the actual and counterfactual trends was analyzed with Student-*t*-test for statistical significance. Analysis was also done for the following secondary endpoints: (i) Duration – proportion of IH events with duration 0–10 s, 10–20 s, and >20 s; (ii) Severity - proportion of IH events in mild category (SpO_2_ range 85%–89%), moderate category (SpO_2_ range 80%–84%), and severe category (SpO_2_ range <80%); (iii) Time spent in hypoxemia (Hours); (iv) Cumulative time spent in hypoxemia (SpO_2_ <90%); (v) Comparison of IH events in infants exposed and unexposed to ACS.

## Results

**Figure 1:**
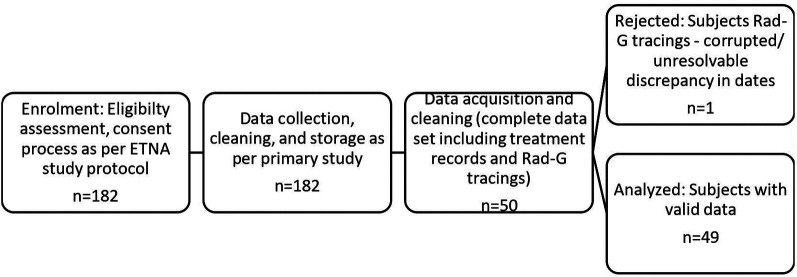
Study flow diagram. A total of 182 infants were eligible for the parent study. However, only 50 had the continuous pulse oximetry required for this study. One of these had corrupted data and was excluded, and therefore the final eligible number was 49.

A total of 49 participants enrolled in the implementation phase 2 of the parent study had complete data available for enrollment and constituted the sample size for the IH study. The monitoring of the babies using the study pulse oximetry gadget was at the discretion of the attending physicians and nurses. A total of 786 patient days (14,502.73 h) of observation was available for participants aged between 26 and 38 weeks and 3 days. The infants were monitored for average blocks of 23 h per day. Caffeine was stopped when infants attained 34 weeks gestational age (Figure 1).

One infant received surfactant treatment, and two infants received CPAP respiratory support soon after birth. None of the study participants needed escalation of care nor was diagnosed with any chronic complication of prematurity such as BPD or retinopathy. The baseline characteristics of the study population and the main outcomes are summarized in Tables 1, 2 respectively.

**Table 1:** Characteristics of the study subjects.

Characteristic	Value
Mothers no. (%)	47 (100)
Age, years median (IQR)	28 (7)
Antenatal steroids, No. (%)	28 (63.6%)
Caesarian section delivery, No. (%)	26 (55.3%)
<28 weeks	0
28–<32 weeks	13 (27.7)
≥32 weeks	13 (27.7)
HTN, No. (%)	14 (29.8)
<28 weeks	0
28–<32 weeks	5 (10.6)
≥32 weeks	9 (19.1)
Infection, No. (%)	13 (27.7)
<28 weeks	0
28–<32 weeks	5 (10.6)
≥32 weeks	8 (17)
Singleton gestation, No. (%)	44 (93.6)
Parity, mean	2.7
Infants at birth No. (%)	49 (100)
Gestational age, No. (%)	
<28 weeks	6 (12.2)
28–<32 weeks	29 (59.2)
≥32 weeks	14 (28.6)
Birth weight (g)	
<28 weeks	870.8
28–<32 weeks	1,337.80
≥32 weeks	1,453.60
Chronological age at recruitment, days median (IQR)	
<28 weeks	3 (6)
28–<32 weeks	5 (5)
≥32 weeks	5 (5)
Female, No. (%)	18 (36.7)
Born at study institution, No. (%)	46 (93.9)
Singleton infant, No. (%)	44 (89.8)
Apgar score at 5 min (MEDIAN, IQR)	8 (2)
Temperature, degrees Celcius, median (IQR)	
<28 weeks	36.1 (1.7)
28–<32 weeks	35.8 (1.1)
≥32 weeks	35.7 (0.85)

**Table 2:** List of outcomes.

S/No	Outcome (measurement unit)	Actual slope (unit/week) - Caffeine ceased	Counterfactual slope (unit/week) - Caffeine not ceased	Post-interruption period slope difference (*p*-value)	Remarks: Impact of caffeine cessation on trend of IH events
1	Frequency of IH events (events/hour)	8.3	0.9	0.000	Increased frequency 7.9–49.9 IH events/hour
2	Duration of IH events (% events)				
	a) 0–10 s	−0.7	3.4	0.001	Decreased proportion of events by 16.4%
	b) 10–20 s	0.9	−2.2	0.000	Increased proportion of events by 12.3%
	c) >20 s	−1.7	0.8	0.000	Decreased proportion of events by 9.9%
3	Severity of IH events (% events)				
	a) Mild	−8.2	3.6	0.000	Decreased proportion of events by 46.9%
	b) Moderate	4.3	−0.1	0.000	Increased proportion of events by 17.4%
	c) Severe	3.7	−2.9	0.000	Increased proportion of events by 26%
4	Time in hypoxemia (SpO_2_ <90%)				
	a) Total time (hours/week)	0.2	0.07	0.013	Increased time in hypoxemia per week to 31 h/week
	b) Cumulative time (hours)	36.7	15.1	0.000	Increased total time in hypoxemia by 85.4 h at 38 weeks GA
5	Antenatal corticosteroids				
	a) No exposure (IH events/hour)	2	10	0.031	Increased events in infants without ACS exposure from 10 to 50 events/hour. ACS-exposed infants had a reduction in events
	b) Exposed (IH events/hour)	2	−1		

### Primary outcome: effect of caffeine cessation on frequency of IH events

**Figure 2:**
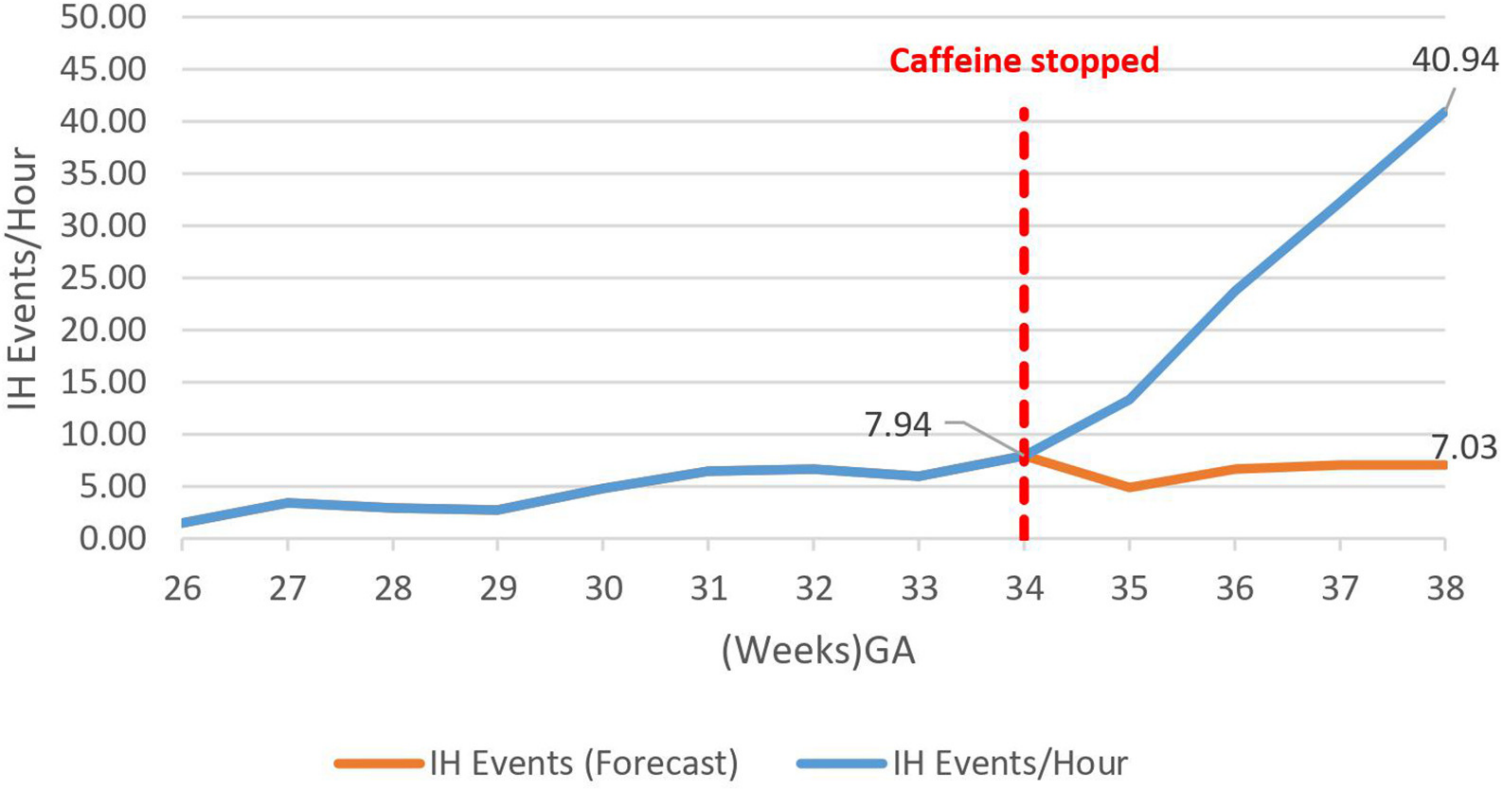
Effect of caffeine cessation on number of intermittent hypoxemia (IH) events. The baseline number of events was 3–8 events per hour while the infants were on caffeine therapy, but these increased exponentially on cessation of therapy reaching 41 events per hour by 38 weeks GA.

Caffeine therapy cessation was associated with a five-fold increase in number of IH events per hour (7.9–40.9 IH events per hour) by 38 weeks gestational age, *p* < 0.01 (Figure 2).

### Secondary outcomes

**Figure 3:**
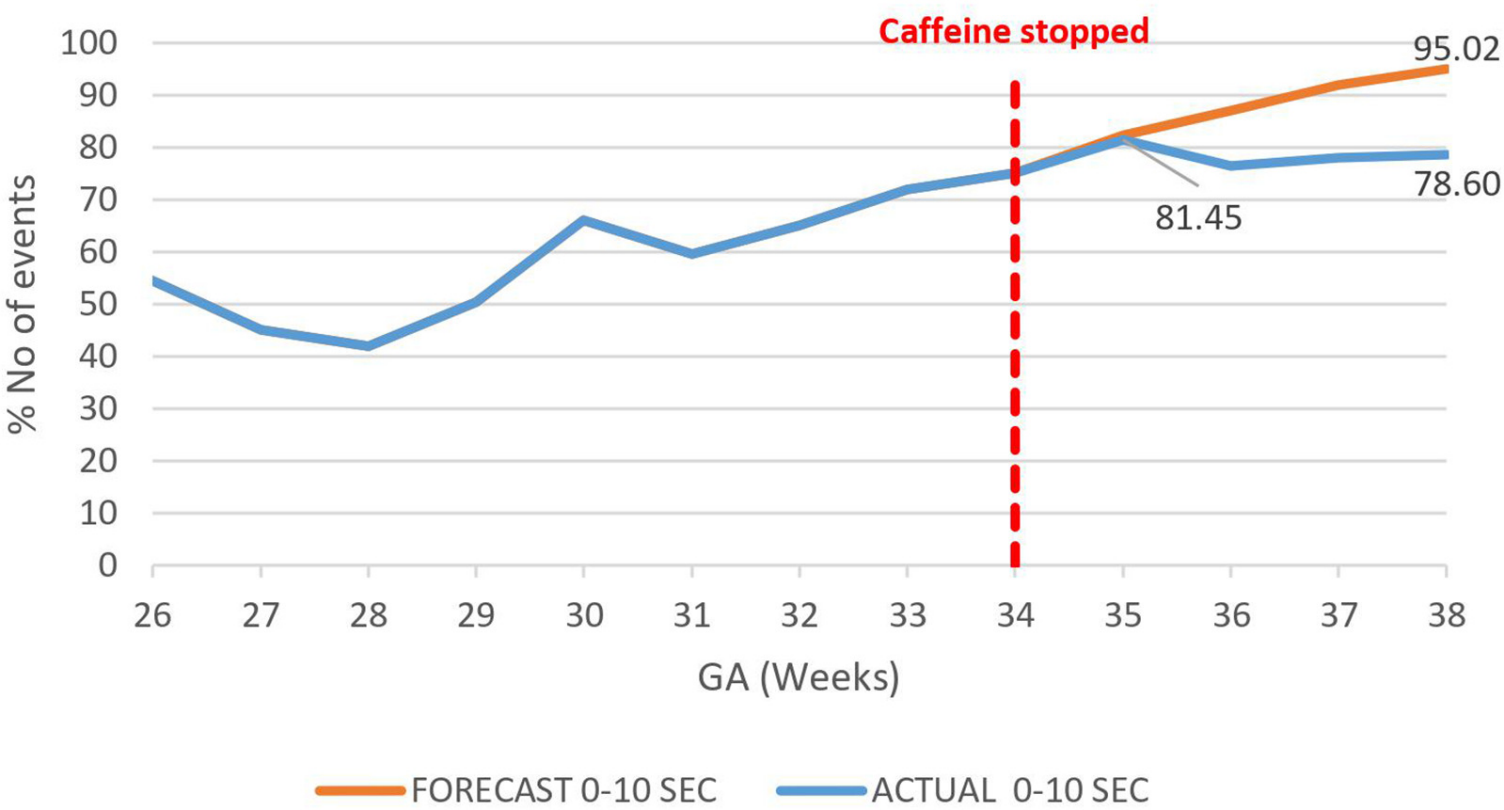
Effect of caffeine therapy cessation on proportion of intermittent hypoxemia (IH) events of 0–10 s duration. The proportion of events of this duration plateaued on cessation of therapy indicating an increase in the events with longer duration.

**Figure 4:**
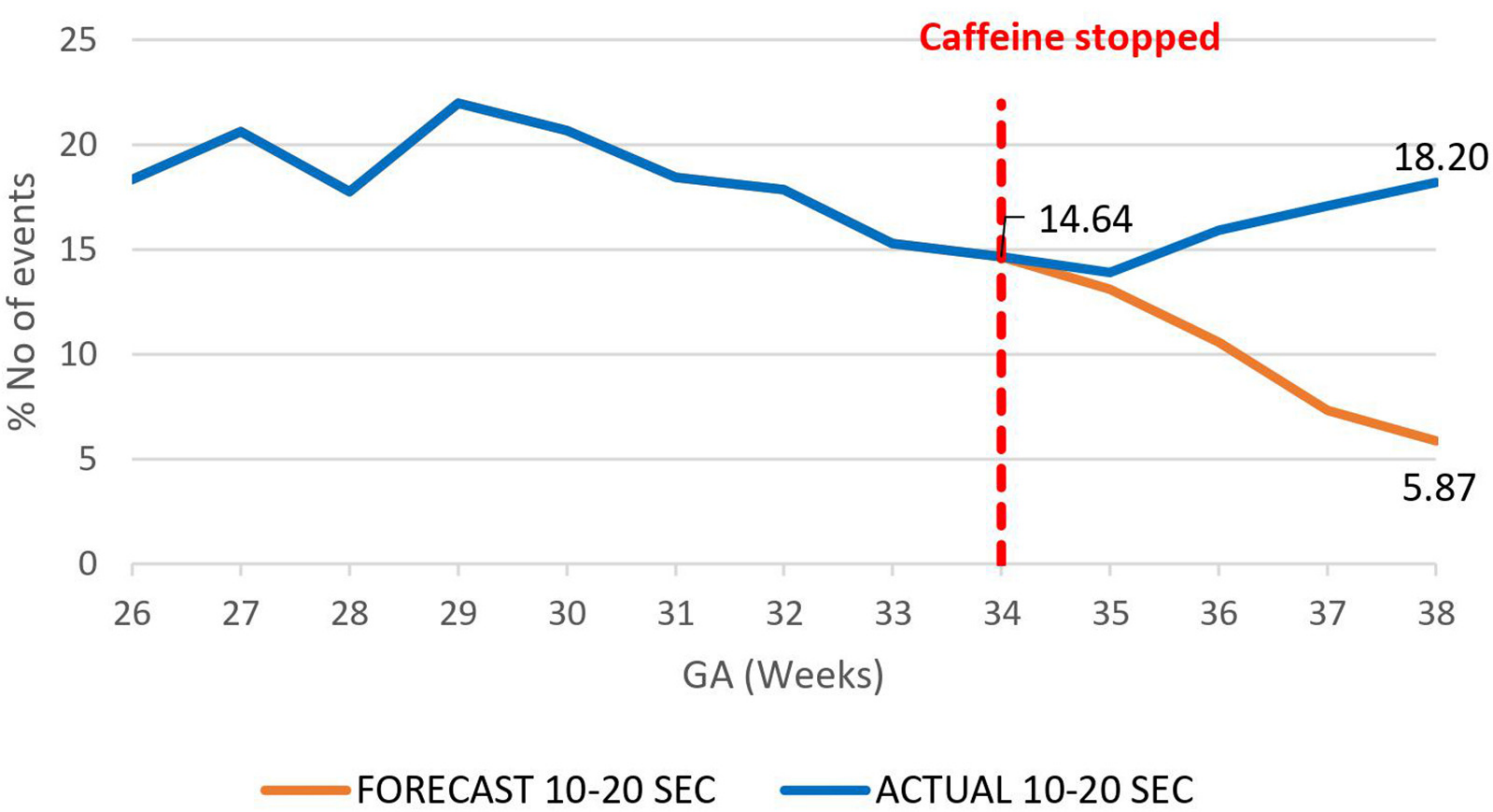
Effect of caffeine therapy cessation on proportion of intermittent hypoxemia (IH) events of 10–20 s duration. The proportion of events stopped decreasing and started increasing in trend one week after cessation of caffeine.

**Figure 5:**
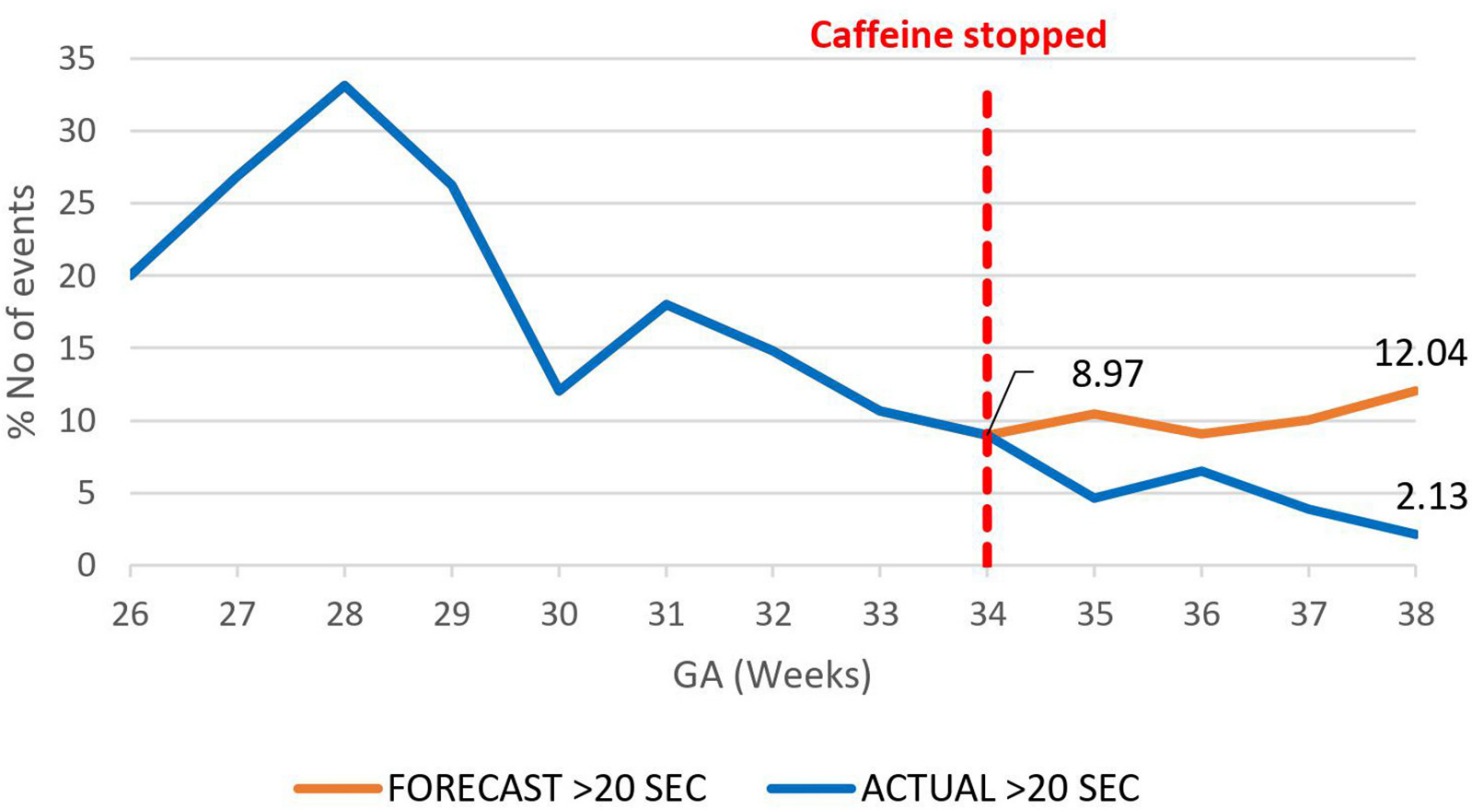
Effect of caffeine therapy cessation on proportion of intermittent hypoxemia events of >20 s duration. The decreasing trend of these events was briefly plateaued after one week and for one week and then resumed a downward trend.

A decrease in the proportion of IH events lasting 0–10 s by 16.4% (95.02%–78.6%) by 38 weeks gestational age was observed, *p* = 0.000785 (Figure 3). However, there was an increase in the proportion of IH events lasting 10–20 s by 12.3% (5.87%–18.20%), *p* < 0.01 (Figure 4) and no effect on the existing downward trend of the proportion of IH events lasting >20 s, *p* < 0.01 (Figure 5).

**Figure 6:**
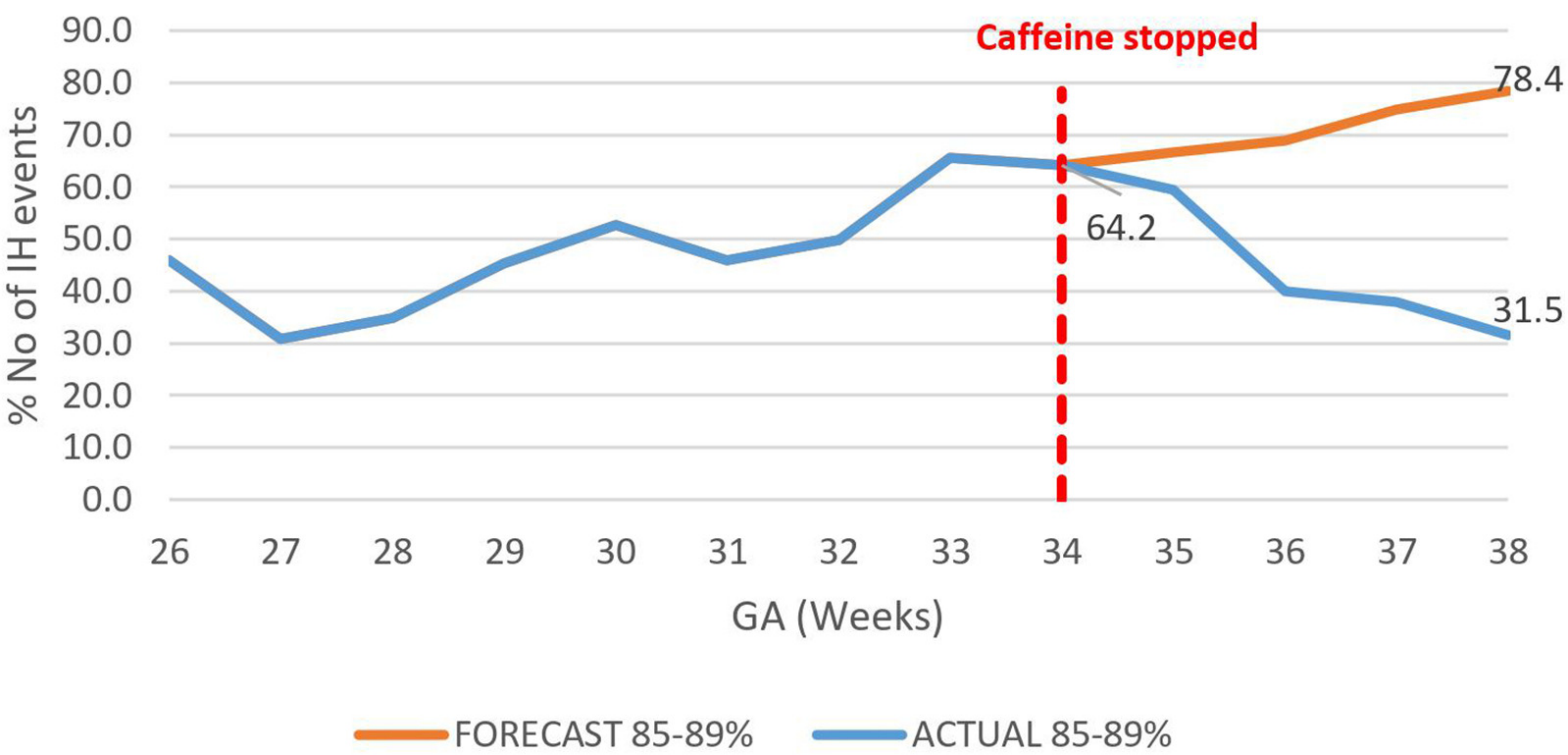
Effect of caffeine therapy cessation on proportion of intermittent hypoxemia events of mild severity (SpO_2_ 85%–89%). There was a change from an increasing to a decreasing trend.

**Figure 7:**
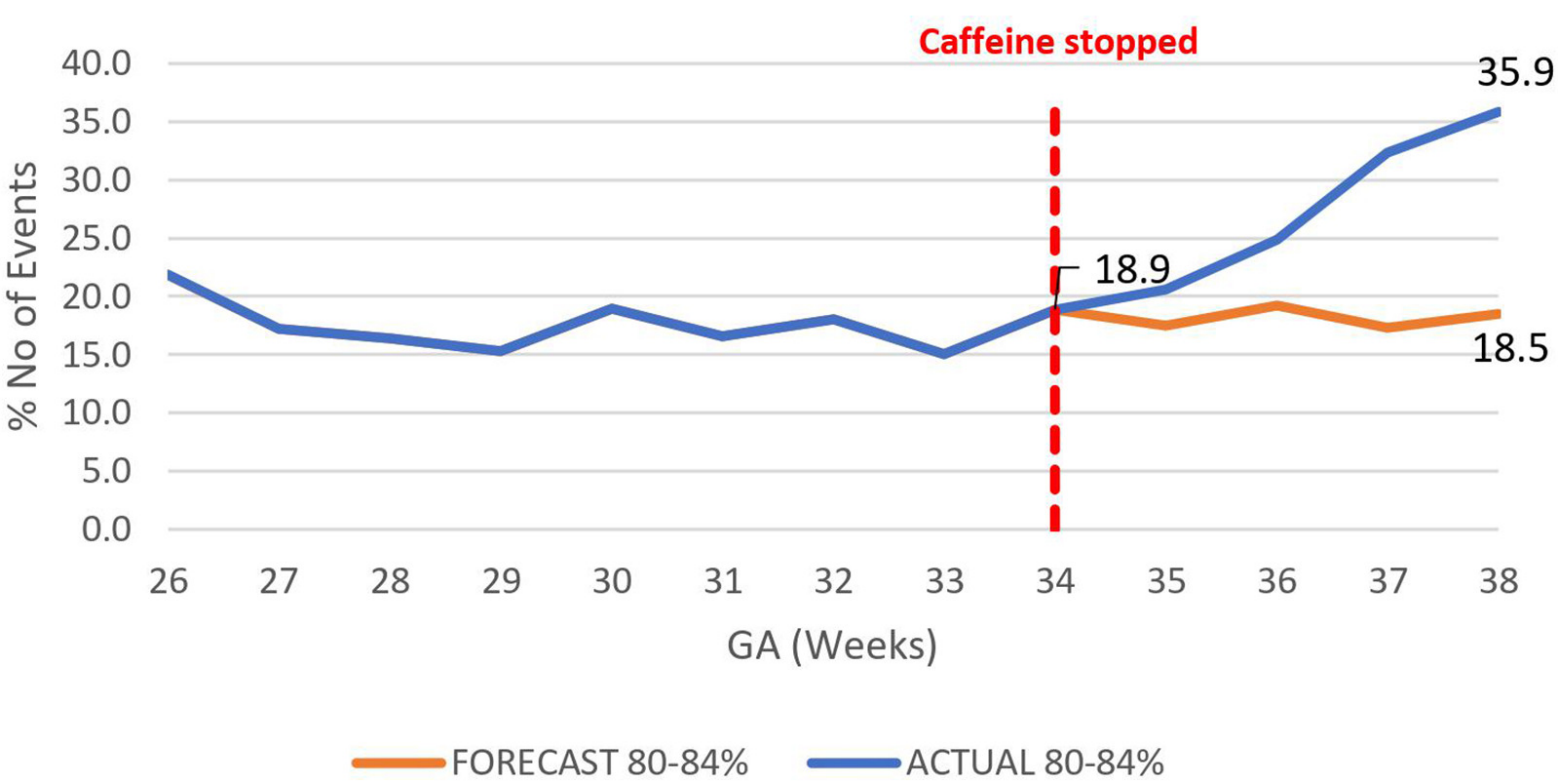
Effect of caffeine therapy cessation on proportion of moderately severe intermittent hypoxemia events (SpO_2_ 80%–84%). There was a change in trend from a plateau of about 17% to an increasing trend reaching 36% by 38 weeks gestational age.

**Figure 8:**
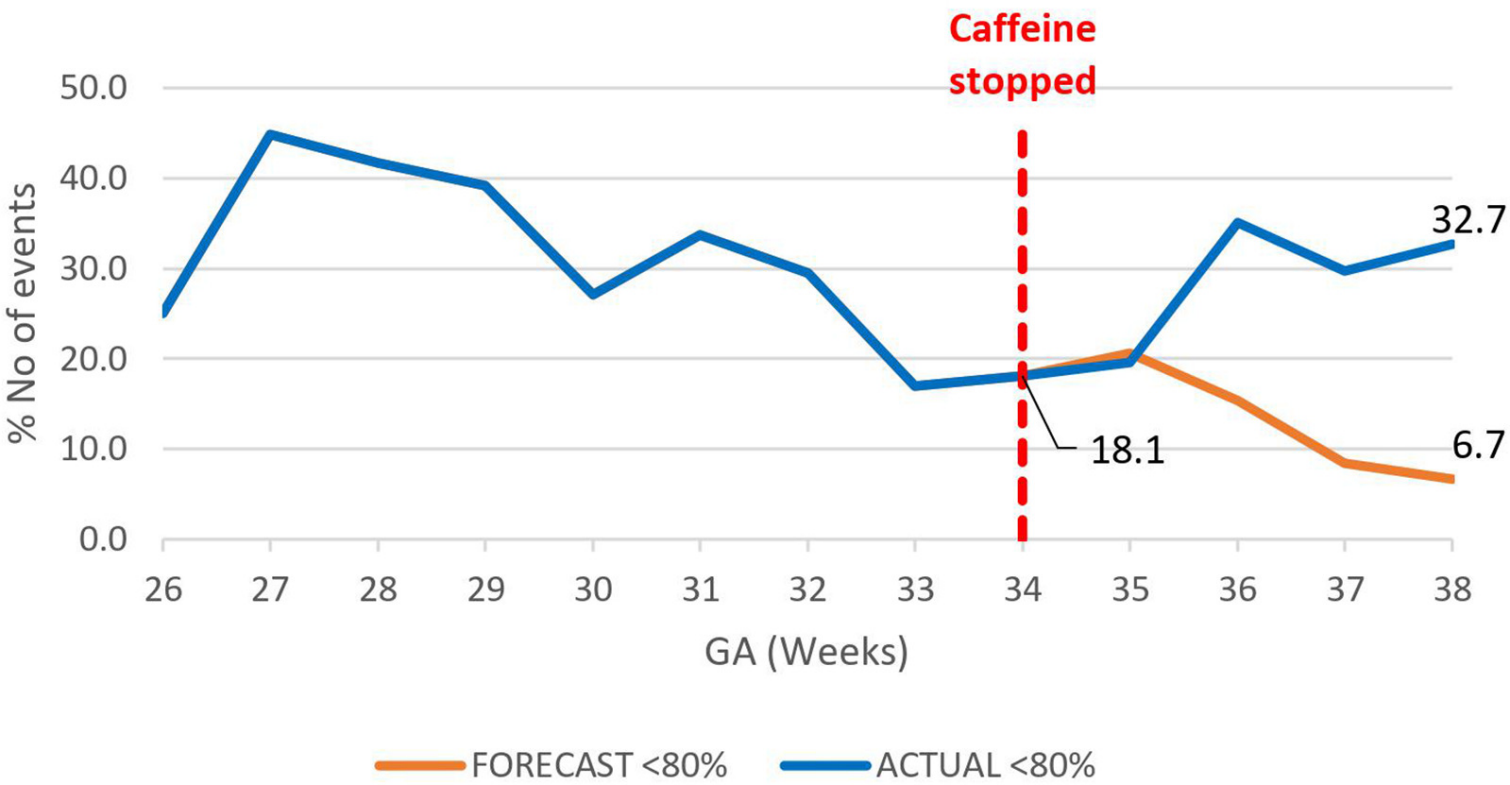
Effect of caffeine therapy cessation on proportion of severe intermittent hypoxemia events (SpO_2_ <80%). The trend changed from a decreasing one to increasing trend.

The overall impact of caffeine cessation on severity of IH events was an increase in severe IH and moderate IH events and a reduction in mild IH events. The mildly severe IH events decreased by nearly half, 46.9% (78.4%–31.5%) *p* < 0.01 (Figure 6), while both the moderately severe and severe IH events increased by 17.4% (18.5%–35.9%), *p* < 0.01 (Figure 7), and by 26% (6.7%–32.7%) respectively, *p* < 0.01 (Figure 8).

**Figure 9:**
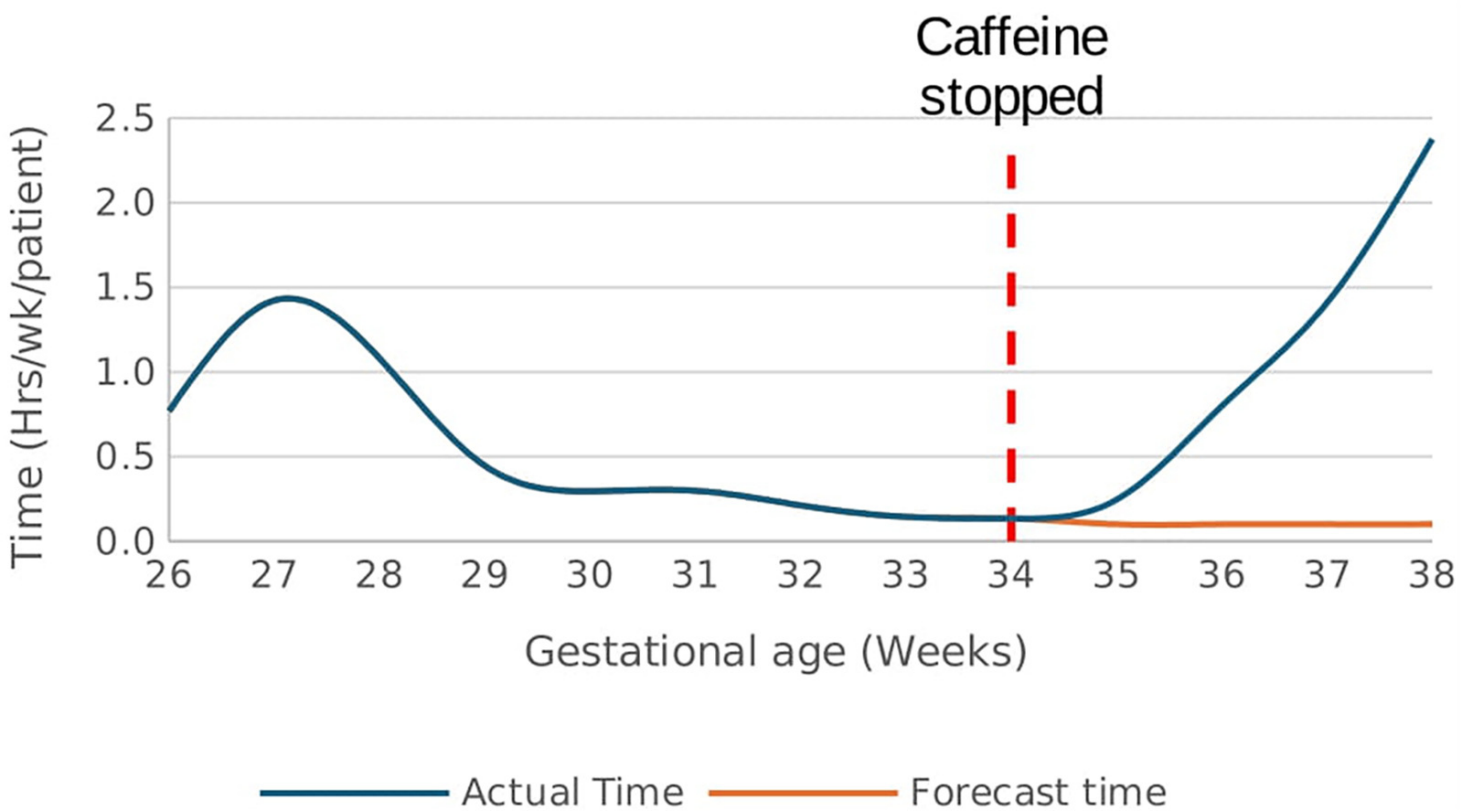
Effect of caffeine therapy cessation on the time (hours/week) spent by infants in hypoxemia (SpO_2_ <90%). There was a drastic increase in the time spent in hypoxemia after cessation of therapy.

**Figure 10:**
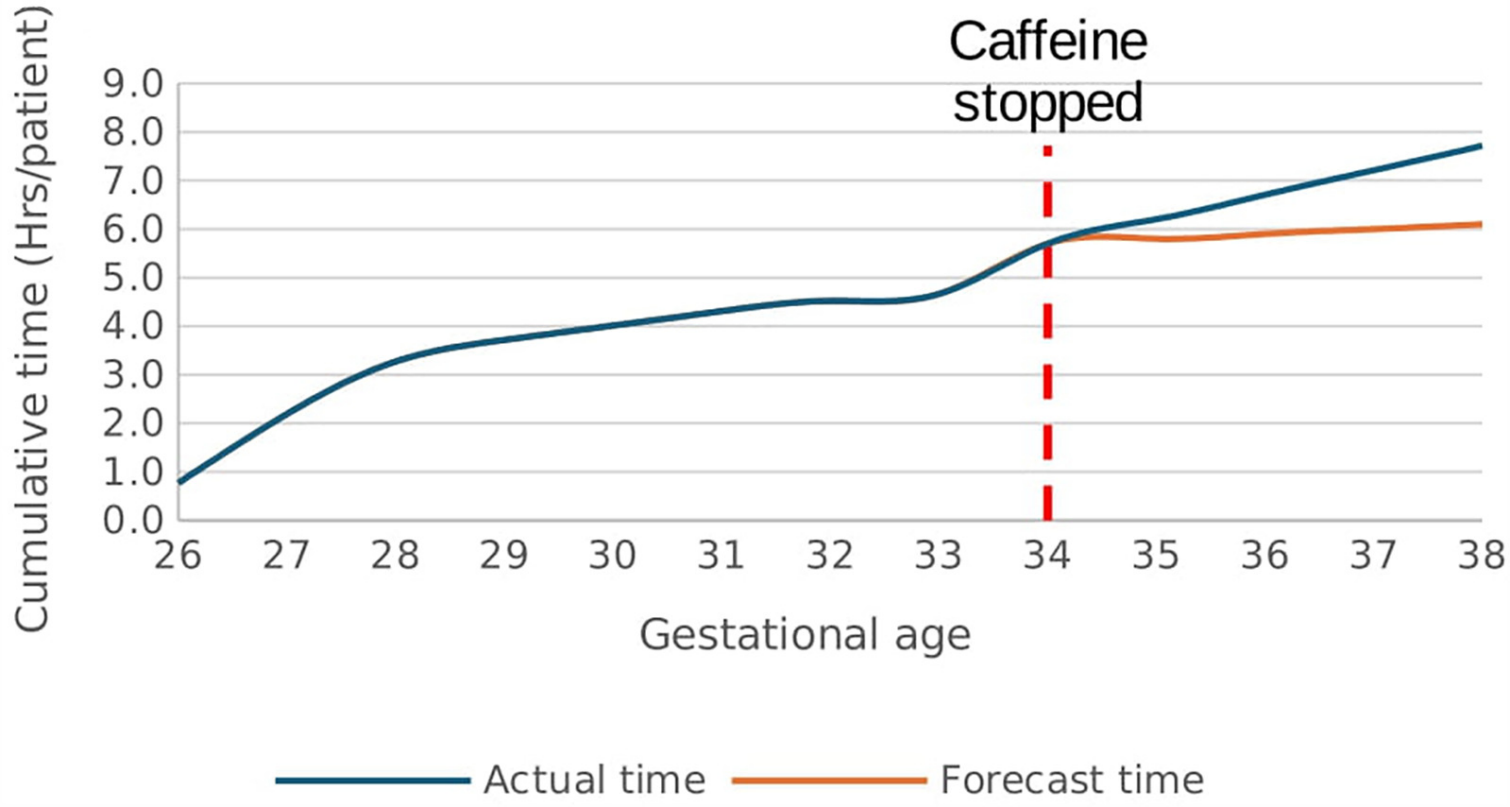
Effect of caffeine therapy cessation on cumulative time spent by infants in hypoxemia (SpO_2_ <90%). The infants had a greater time spent in hypoxemia when therapy was stopped compared with if therapy continued.

There was a rapid increase in the rate of time spent in hypoxemia from a baseline rate of 0.1 h/week/patient after caffeine cessation at 34 weeks GA reaching a rate of 2.4 h/week/patient at 38 weeks GA *p* < 0.01, Figure 9. In effect, the cumulative time spent in hypoxia increased by 1.6 h/patient above the forecast time by end of 38 weeks gestational age *p* < 0.01, Figure 10. This increasing trend was also demonstrated on sub-analysis of data by severity of IH events.

**Figure 11:**
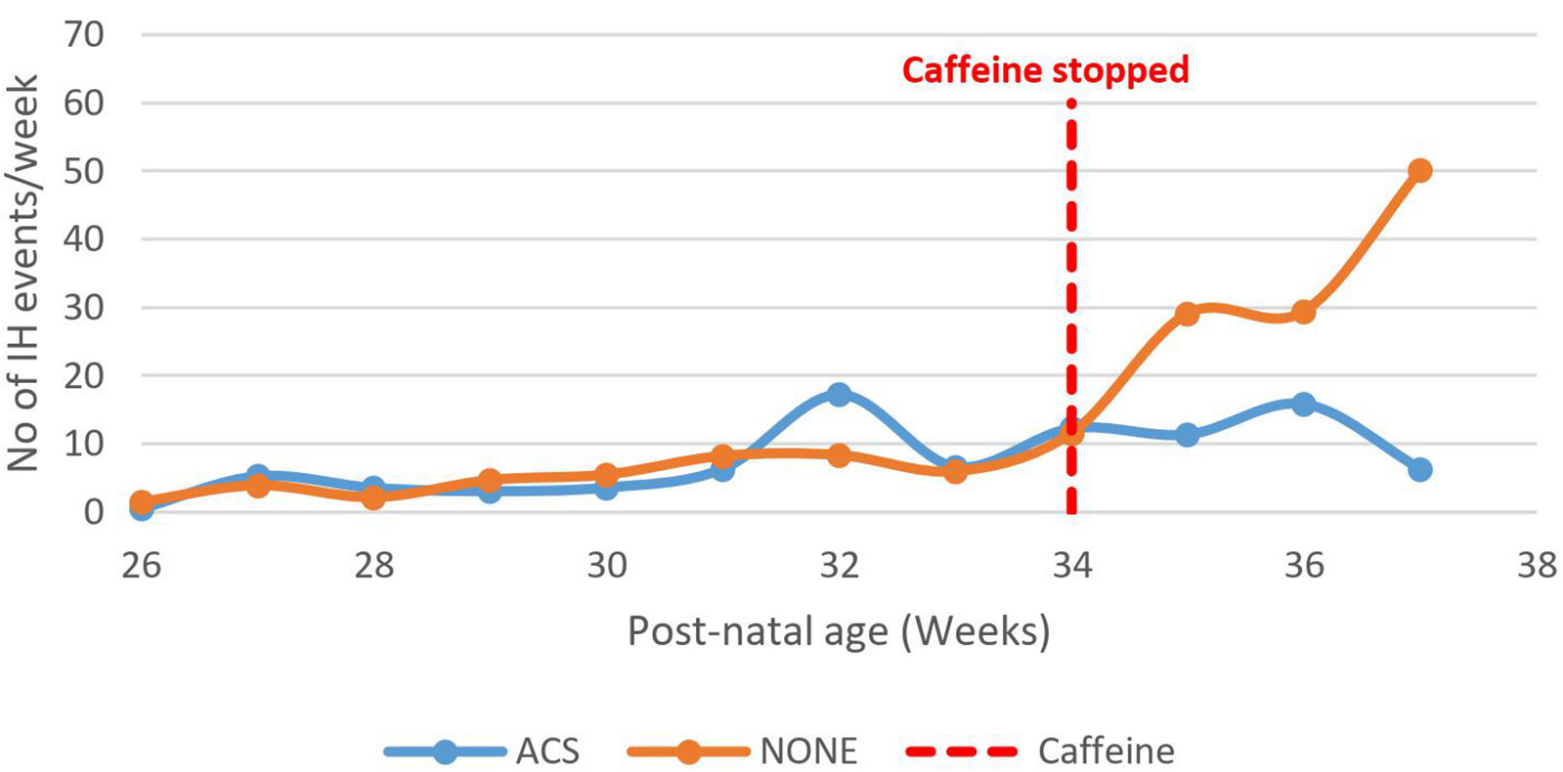
Effect of caffeine therapy cessation on the number of intermittent hypoxemia events among babies exposed and those not exposed to maternal antenatal steroids. Both groups of babies had similar patterns during therapy. The babies whose mothers received antenatal steroids did not experience a rise in intermittent hypoxia events on cessation of therapy awhile those that were not exposed experienced a rapid rise in the number of events.

The number of IH events increased among infants who were not exposed to ACS compared to those exposed, *p* = 0.03 (Figure 11).

## Discussion

Current guidelines recommend caffeine citrate as the drug of choice for prevention of AoP in all infants born prior to 34 weeks GA until 34–36 weeks with no clinically apparent apnea for 5–7 days and was the practice in the tertiary care NBU (19, 20). The IH events of a low-risk phenotype existed during caffeine therapy at a rate of 2–8 IH events per hour, lasting 0–10 s and with nadir SpO_2_ in the mild 85%–89% range. Upon cessation of caffeine therapy, the IH events were transformed to high-risk phenotype with a sharp and steady increase in frequency, duration, and severity. While existing studies indicate that continuation of therapy maintains the low risk IH events, this study demonstrates that interruption of therapy transforms these events to the high-risk type (1, 3, 4, 6, 15). The clinical significance of this finding remains to be evaluated.

The proportion of IH events with prolonged duration of greater than 20 s were also detected in these infants during caffeine therapy. Although there was a declining trend, the absolute number of these IH events increased due to the overall increase in the number of IH events. This highlights the importance of the need for high quality monitoring devices since such events will not be identified and considered during evaluation for caffeine cessation at 34 weeks (6, 15).

The study participants experienced IH events while on caffeine therapy contributing to a cumulative time spent in hypoxemia of 5.7 h/patient by 34 weeks gestational age. Cessation of caffeine at 34 weeks increased the cumulative time spent in hypoxemia to 7.7 h/patient hours by 38 weeks GA compared with 6.1 h/patient forecasted with continued therapy. This is an additional 1.6 h/patient (26.2%) more time in hypoxemia. The clinical significance of this observation is unclear, and further studies may be useful. Using appropriate technologies to detect apnea and IH events could optimize time spent in the recommended target range of 90%–95% and potentially avert adverse neonatal neurologic outcomes associated with high-risk IH events (11, 14).

Most of the preterm infants in this study were born between 28 and 32 weeks gestational age and were on the rising phase or peak of IH events at 34 weeks. Therefore, withdrawal of caffeine citrate therapy may have unmasked this natural trend in keeping with the natural history patterns previously described (6, 8). Exposure to ACS seemed to be protective with a declining trend of IH events after cessation of caffeine citrate and complements the findings of an earlier study by Abu Jawdeh et al. (15). ACS are postulated to act through improved maturation of the respiratory system, CNS breathing control centers, as well as reducing neonatal co-morbidities that by interrelated mechanisms contribute to adverse outcomes (11, 15).

Factors such as maturity and genetic variation contribute to the variability in clinical responses to caffeine therapy in preterm infants; therefore, the optimal dose is unknown (20, 21). There is great variability in institutional approaches concerning caffeine citrate dosage, ranging from a low-dose to a high-dose approach (up to 40 mg/kg loading dose at birth followed by 20 mg/kg/day maintenance) (20). However, caffeine citrate has a wide therapeutic range and is generally safe for prolonged use among preterm infants (20, 21).

Sensitive and continuous monitoring equipment are often unavailable or inadequate in low-resource low- and middle-income settings due to cost and human resource constraints and further is hampered by the large numbers of patients in NBUs necessitating monitoring (2, 6, 25). In the absence of adequate monitoring, the continuation of caffeine therapy beyond 34 weeks may be beneficial in prevention of the potential detrimental effects of hypoxemia. A well-designed randomized controlled trial is needed to investigate the usefulness and safety of this approach as well as the clinical significance of these findings.

## Study limitations

This was a retrospective secondary analysis of data collected from neonatal infants monitored for AoP. A major limitation was the unavailability of continuous monitoring data of all participants throughout their NBU stays during the study period. Therefore, selected infants were monitored for variable and intermittent durations and the data available from individual patients could not provide a linear follow-up.

The selection of infants who were monitored was at the discretion of the NBU attending physicians, much like it is in practice, and was based on the perceived risk of AoP as per the objectives of the overall study. Potential bias for selection for sicker babies may not be excluded. Given this study was not longitudinal, other important clinical outcomes and the clinical significance of the findings could not be evaluated.

The definition of IH and cut-off durations and SpO_2_ levels are variable in literature. The definitions utilized in this study were selected *a priori* to allow comparisons to similar studies.

## Conclusions

IH and IH events in apnea range occur in preterm infants born before 34 weeks gestational age receiving low-dose caffeine citrate therapy for AoP and contribute to a significant time spent in hypoxemia. The cessation of caffeine therapy at 34 weeks is associated with a dramatic increase in high-risk IH events and total time spent by the infants in hypoxemia. ACS exposure may be protective against this transformation of IH events.

## Data Availability

The datasets presented in this article are not readily available because Consent of the PI of the original ETNA study will be required. Requests to access the datasets should be directed to william.macharia@aku.edu.
